# Nanoscale dynamics of cholesterol in the cell membrane

**DOI:** 10.1074/jbc.RA119.009683

**Published:** 2019-07-03

**Authors:** Kerstin Pinkwart, Falk Schneider, Martyna Lukoseviciute, Tatjana Sauka-Spengler, Edward Lyman, Christian Eggeling, Erdinc Sezgin

**Affiliations:** ‡ MRC Human Immunology Unit, MRC Weatherall Institute of Molecular Medicine, University of Oxford, Oxford OX3 9DS, United Kingdom; § Radcliffe Department of Medicine, Weatherall Institute of Molecular Medicine, University of Oxford, Oxford OX3 9DS, United Kingdom; ¶ Departments of Physics and Astronomy and Chemistry and Biochemistry, University of Delaware, Newark, Delaware 19716; ‖ Institute of Applied Optics and Biophysics, Friedrich-Schiller-University Jena, Max-Wien Platz 4, 07743 Jena, Germany; ** Leibniz Institute of Photonic Technology e.V., Albert-Einstein-Straße 9, 07745 Jena, Germany

**Keywords:** cholesterol, fluorescence correlation spectroscopy (FCS), plasma membrane, membrane biophysics, membrane lipid, membrane structure, Bodipy-cholesterol, diffusion, hindered diffusion, membrane asymmetry, model membranes

## Abstract

Cholesterol constitutes ∼30–40% of the mammalian plasma membrane, a larger fraction than of any other single component. It is a major player in numerous signaling processes as well as in shaping molecular membrane architecture. However, our knowledge of the dynamics of cholesterol in the plasma membrane is limited, restricting our understanding of the mechanisms regulating its involvement in cell signaling. Here, we applied advanced fluorescence imaging and spectroscopy approaches on *in vitro* (model membranes) and *in vivo* (live cells and embryos) membranes as well as *in silico* analysis to systematically study the nanoscale dynamics of cholesterol in biological membranes. Our results indicate that cholesterol diffuses faster than phospholipids in live membranes, but not in model membranes. Interestingly, a detailed statistical diffusion analysis suggested two-component diffusion for cholesterol in the plasma membrane of live cells. One of these components was similar to a freely diffusing phospholipid analogue, whereas the other one was significantly faster. When a cholesterol analogue was localized to the outer leaflet only, the fast diffusion of cholesterol disappeared, and it diffused similarly to phospholipids. Overall, our results suggest that cholesterol diffusion in the cell membrane is heterogeneous and that this diffusional heterogeneity is due to cholesterol's nanoscale interactions and localization in the membrane.

## Introduction

Cholesterol plays a pivotal role in eukaryotic cellular membranes both structurally and functionally (1–3). Besides its involvement in signaling (4–8), it considerably shapes the molecular architecture of the plasma membrane (9, 10). However, due to the challenge of performing high resolution measurements on small and fast molecules in cells, molecular-level knowledge is limited regarding specific mechanisms involving cholesterol. To better understand how cholesterol influences cellular signaling, it is essential to directly observe the dynamics of cholesterol in the plasma membrane.

Fluorescence microscopy and spectroscopy are powerful tools for studying molecular dynamics in living cells (11). Visualization of cellular cholesterol with these approaches is usually achieved using fluorescently-labeled cholesterol analogues (11). These analogues, however, may not reflect the native behavior of cholesterol because the cholesterol and the fluorescent tag are of similar size, a design consideration for all fluorescent lipid analogues. One important feature of robust lipid analogues is that they partition into membrane regions like their unlabeled counterparts. Ordered membranes (or nanodomains) are enriched with saturated lipids, some of which (such as sphingomyelin) have preferential cholesterol binding (12). Therefore, the cholesterol analogues that are enriched in the ordered plasma membrane domains are considered to be reliable mimics of native cholesterol (2, 9). Another important criterion for the reliability of fluorescent lipid analogues is proper cellular trafficking into subcellular structures. BODIPY-labeled cholesterol, for instance, has shown a great potential to mimic cholesterol in terms of partitioning, dynamics, and cellular localization (13–17). Despite its ordered domain partitioning, interestingly, recent reports suggested an order of magnitude faster diffusion for cholesterol analogues compared with phospholipids (18, 19). This finding brings a conundrum; how can cholesterol move faster in the cell membrane while being enriched in more ordered (packed) membrane environments? To answer this, studies aiming at revealing the nanoscale cholesterol diffusion in the cell membranes and its determinants are needed.

Nanoscale behavior of molecules is not readily accessible with conventional methodologies. The investigation of nanoscale dynamics of specific molecules in the membrane has been hampered by the complex, heterogeneous, and hierarchical structure of the cell membrane. Moreover, nanoscale interactions occur at very fast temporal and small spatial scales, which presents additional experimental challenges. Therefore, a complete understanding of nanoscale molecular behavior requires both advanced imaging techniques and well-defined model membrane systems. Here, we applied advanced imaging and spectroscopy approaches on *in vitro* (model membranes) and *in vivo* (live cells and embryos) membranes to elucidate the nanoscale diffusion dynamics of cholesterol in biological membranes. We compared the diffusion of cholesterol with that of phospholipids as well as of cholesterol analogues carrying acyl chains. The experimental measurements were complemented by *in silico* Monte Carlo and Molecular Dynamics simulations. The data show that cholesterol diffuses faster than phospholipids in cellular membranes, particularly in live membranes. Furthermore, membrane domain partitioning does not influence the nanoscale diffusion, whereas nanoscale interactions and localization in the plasma membrane significantly influence the diffusional dynamics of cholesterol.

## Results and discussion

**Figure 1.**
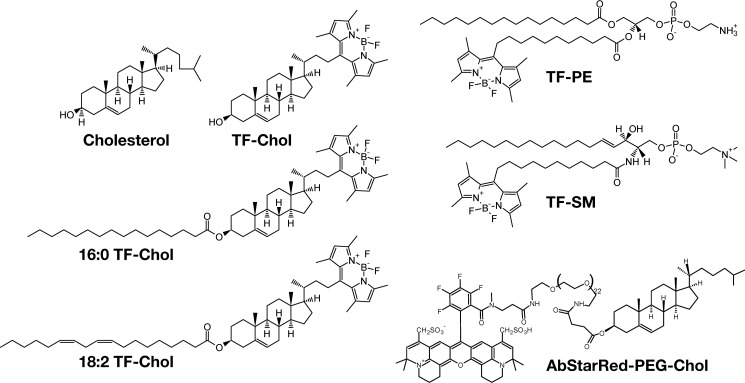
**Structures of fluorescent lipid analogues used in this study.**

To understand cholesterol dynamics at the nanoscale in biomembranes, we first compared the diffusion dynamics between various cholesterol analogues and phospholipid analogues (Fig. 1). Fluorescent lipid labeling is generally a challenging issue due to the comparable sizes of the lipids and the fluorescent tags. It is particularly a major problem for cholesterol labeling due to its comparably smaller size (20). A few intrinsically fluorescent analogues (21–23) and BODIPY-labeled (trademarked as TopFluor® (TF)) cholesterol were shown to mimic cholesterol behavior in model and cell membranes (13, 14, 16, 24, 25). Similarly a few NBD-labeled cholesterol were reported to mimic certain properties of cholesterol (26, 27). Recently, cholesterol was also tagged with different fluorophores (*e.g.* Abberior Star Red (AbStarRed)) via a PEG linker between the fluorophore and sterol moiety (28) to avoid the fluorophore interaction with the membrane (29). Here, we employed TF-Chol^2^ and AbStarRed-PEG-Chol as cholesterol analogues. We also used TF-Chol attached to a saturated (16:0) and an unsaturated (18:2) acyl chain to study whether the acyl chains make cholesterol behave similar to phospholipids in the membrane. In addition, we used phosphatidylethanolamine (PE) and sphingomyelin (SM) tagged with TopFluor (Fig. 1) to directly compare the cholesterol diffusion with other lipid species.

### Diffusion of cholesterol analogues compared with phospholipid analogues in model membranes

**Figure 2.**
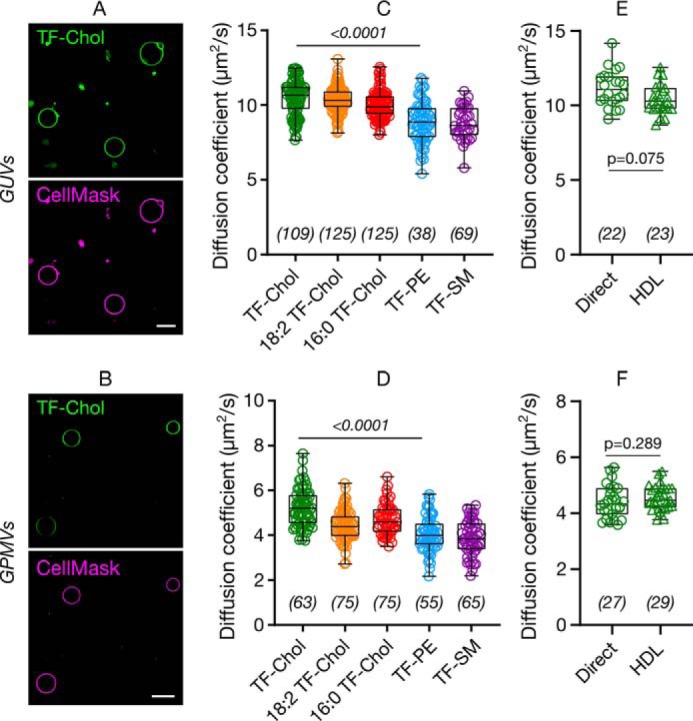
**Diffusion of cholesterol compared with phospholipids in model membranes.** Confocal images of (*A*) GUVs and (*B*) GPMVs labeled with TF-Chol (*green*) and CellMask (*magenta*), which labels the membrane. *Scale bars* are 10 μm. Diffusion of cholesterol and phospholipid analogues in (*C*) GUVs and (*D*) GPMVs. TF-Chol diffusion in (*E*) GUVs and (*F*) GPMVs with direct labeling or via HDL particles. Data are shown as box-and-whisker plot showing median, first and third quartiles, and all the data points. Number of data points are indicated on the graphs.

Previous reports suggested fast diffusion of TF-Chol (≈3 μm^2^/s) in the plasma membrane of live cells (18, 19). To tackle how cholesterol moves compared with phospho- and sphingolipids in the cell membrane systematically, we used simple artificial model membranes (giant unilamellar vesicles; GUVs), cell-derived vesicles (giant plasma membrane vesicles; GPMVs), live mammalian cells, and zebrafish embryos. First, we compared whether an acyl chain attachment changes the cholesterol mobility. To this end, we compared the diffusion of TF-Chol with TF-Chol bearing either saturated or unsaturated acyl chains (16:0 TF-Chol and 18:2 TF-Chol, respectively) (Fig. 1). We stained the synthetic GUVs and cell-derived GPMVs with low amounts of the fluorescent analogues and Cell Mask deep red for membrane visualization (Fig. 2, *A* and *B*). Then, we performed fluorescence correlation spectroscopy (FCS) to obtain the diffusion coefficients for each fluorescent analogue. In GUVs, acyl-chain carrying cholesterol analogues did not show notably different diffusion (D_16:0_ = 10.0 ± 0.9 μm^2^/s; D_18:2_ = 10.3 ± 0.8 μm^2^/s) compared with TF-Chol (D_TF-Chol_ = 10.5 ± 1.2 μm^2^/s) (Fig. 2*C*). TF-PE and TF-SM showed slightly slower (≈1.2 times) diffusion with diffusion coefficients of 8.9 ± 1.4 and 8.8 ± 1.1 μm^2^/s, respectively (Fig. 2*C*), compared with TF-Chol. In GPMVs isolated from Chinese hamster ovary (CHO) cells, addition of an acyl chain only slightly changed the diffusion coefficient of the TF-Chol analogues (Fig. 2*D*). There was, however, a clear difference between TF-Chol and TF-PE or TF-SM (Fig. 2*D*; D_TF-Chol_ = 4.9 ± 0.7 μm^2^/s compared with D_TF-PE_ = 4.0 ± 0.8 μm^2^/s, D_TF-SM_ = 3.9 ± 0.8 μm^2^/s). The lipid analogues were generally slower in GPMVs compared with GUVs and in both model systems TF-Chol diffusion was ≈1.2 times faster than TF-PE or TF-SM. This difference was small but reproducible (Fig. S1).

Cell membranes can be labeled with cholesterol analogues via different approaches such as direct labeling through incubation of the sample with pure TF-Chol or with high and low density lipoproteins (HDL, LDL) particles carrying fluorescent cholesterol (16, 30, 31). The latter is of great efficiency because these particles primarily manage cholesterol transfer in the body. It should be taken into account that the delivery methods may change how the cholesterol is incorporated into the cell membranes that might affect its subsequent diffusion. To this end, we also measured the diffusion of TF-Chol in GUVs and GPMVs where vesicles are labeled either directly by adding the TF-Chol to the vesicle suspension or via HDL particles carrying TF-Chol. In both GUVs and GPMVs, cholesterol diffusion via direct labeling and via HDL particles yielded the same diffusion coefficients (Fig. 2, *E* and *F*).

### Diffusion of cholesterol analogues compared with phospholipid analogues in live membranes

**Figure 3.**
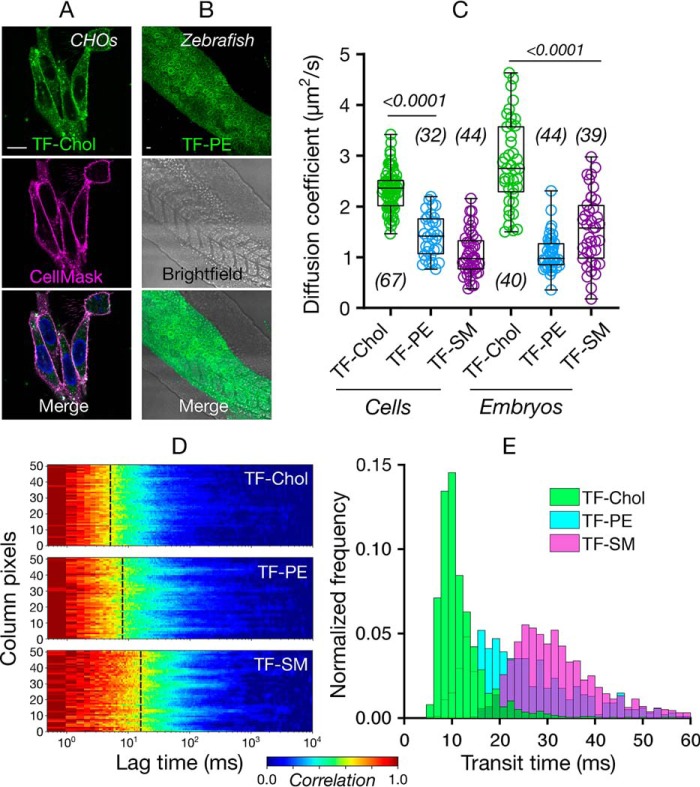
**Diffusion of cholesterol compared with phospholipids *in cellulo* and *in vivo*.** Confocal images of (*A*) CHO cells and (*B*) zebrafish embryos (24 h post fertilization) labeled with fluorescent lipid analogues (*green*) and Cell Mask Deep Red (*magenta*). *Scale bars* are 10 μm. *C,* diffusion coefficient of the fluorescent lipid analogues in CHO cells and zebrafish embryos. *D,* representative sFCS carpets for fluorescent lipid analogues in CHO cells. *E,* histogram of transit diffusion times of the fluorescent lipid analogues obtained from the sFCS data. Data are shown as box-and-whisker plot showing median, first and third quartiles, and maximum and minimum values. Number of data points are indicated on the graphs.

Next, we investigated diffusion of cholesterol in live membranes. We tested the diffusion of cholesterol, phospholipid, and sphingomyelin analogues in CHO cells as an *in cellulo* model for mammalian cells (Fig. 3*A*) and in zebrafish embryos as an *in vivo* model (Fig. 3*B*, see Fig. S2 for details of FCS measurements). In both of these systems we observed markedly faster diffusion of TF-Chol compared with TF-PE or TF-SM (Fig. 3*C*). In CHO cells, the diffusion coefficient was 2.4 μm^2^/s for TF-Chol, 1.4 μm^2^/s for TF-PE, and 1.1 μm^2^/s for TF-SM. In zebrafish embryos, the diffusion coefficient was 2.8 μm^2^/s for TF-Chol, 1.1 μm^2^/s for TF-PE, and 1.5 μm^2^/s for TF-SM. Overall, we observed ≈2-fold faster diffusion of TF-Chol compared with TF-labeled phospholipid and sphingolipid analogues *in vivo*. In model membranes, this difference was ≈1.2 times, which suggests processes in living membranes lead to slower diffusion of phospholipids and sphingolipids compared with cholesterol.

Although the diffusion data obtained with point FCS is extremely informative, it is limited to a diffraction-limited spot and may overlook spatial heterogeneity. The remedy for this is scanning FCS (sFCS), which reports on molecular diffusion along a line (≈5 μm long, ≈50 pixels), thus captures the spatial diffusional heterogeneity in the plasma membrane and better probes the nanoscale dynamics (32). Moreover, sFCS yields multiple curves (as many as the number of pixels) per measurement, which increases the statistical accuracy of the measurements (28). We, thus, performed sFCS to study the diffusion of fluorescent lipid analogues in the cell membrane with more accurate spatial sampling. sFCS carpets (which are color-coded FCS curves, red showing the maximum correlation, blue showing the minimum correlation) on live CHO cells show clear fast diffusion of TF-Chol compared with TF-PE and TF-SM (yellow parts corresponds to the transit diffusion time, Fig. 3*D*). Histogram of the transit times obtained from the sFCS data confirms the fast diffusion of TF-Chol, slower diffusion of TF-PE, and slowest diffusion of TF-SM (Fig. 3*E*).

### Diffusion of fluorescent lipid analogues versus their membrane domain partitioning

**Figure 4.**
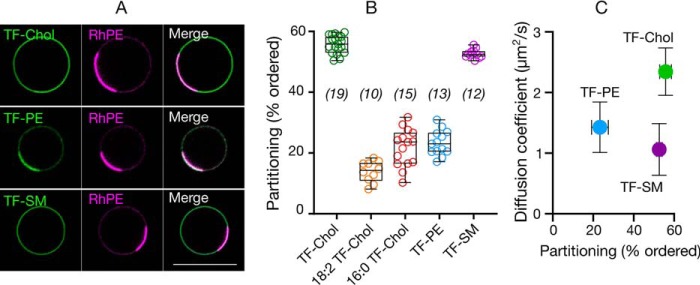
**Relationship between diffusion in cells and analogue partitioning in phase-separated GPMVs.** *A,* confocal images of TF-Chol, TF-PE, and TF-SM partitioning in phase separated GPMVs with rhodamine-PE (*RhPE*) as disordered phase marker. *Scale bar* is 10 μm. *B,* quantification of ordered domain partitioning of fluorescent lipid analogues from the confocal images. *C,* correlation between ordered domain partitioning in GPMVs and diffusion of fluorescent lipid analogues in cells. Data are shown as box-and-whisker plot showing median, first and third quartiles, and all the data values. Number of data points are indicated on the graphs.

We next set out to determine which aspects of living membranes cause faster diffusion of cholesterol in cells or, more precisely, slower diffusion of phospholipid and sphingolipid analogues. One explanation is differential partitioning into membrane environments of different fluidity and packing. Molecules diffuse more slowly in ordered plasma membrane environments enriched with saturated lipids compared with disordered membranes enriched with unsaturated lipids (33). Coexistence of ordered and disordered domains is observed in model membranes depending on composition and temperature. The most physiological systems where such macroscopic phase separation is observed are GPMVs derived from live cells (34, 35). Therefore, to test whether the discrepancy between the diffusion of the analogues we observe can be attributed to heterogeneous partitioning in the plasma membrane, we correlated partitioning in phase-separated GPMVs with diffusion in cell membranes. TF-Chol and TF-SM partitioned into the ordered domain (≈60%), whereas others predominantly partitioned into the disordered domain (Fig. 4, *A* and *B*). When we compared the ordered domain partitioning with the diffusion properties, we observed no correlation between the diffusion and partitioning (Fig. 4*C*). Therefore, we conclude that the slow diffusion of TF-PE and TF-SM is not related to their partitioning into membrane domains and that the domain incorporation may not correlate strongly with probe diffusion.

### Diffusion of fluorescent lipid analogues versus their nanoscale interactions

Another explanation for the slow diffusion of TF-PE and TF-SM relative to cholesterol in live cell membranes can be nanoscale hindrances to diffusion that affect phospholipids, but not cholesterol. A “hindered diffusion mode” is often reported for various membrane components, but has not been systematically studied for cholesterol. The key to such measurements is to determine how the apparent diffusion coefficient of molecules changes with the size of the observation spot (36). For a molecule undergoing Brownian (or free) diffusion, the apparent diffusion coefficient is independent of the size of the observation spot, whereas for hindered diffusion, the diffusion coefficient varies depending on spot size. The qualitative dependence of the effective diffusion constant on observation volume depends on the underlying diffusion law. It decreases with decreasing observation spot size when molecules are transiently immobilized or trapped or incorporated into slow-moving molecular complexes with sizes below the diffraction limit (37, 38). Conversely, it will increase with decreasing spot size when molecules undergo hop or compartmentalized diffusion in a meshwork-like pattern (39–41).

**Figure 5.**
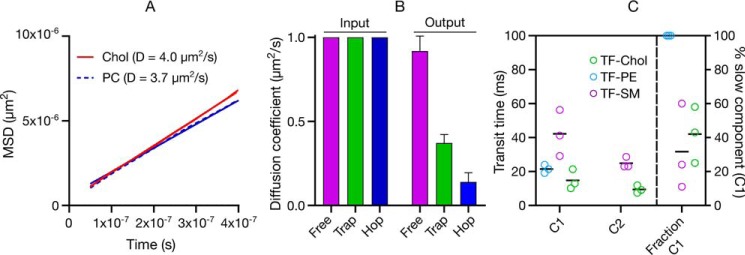
**Diffusion mode of fluorescent lipid analogues.** *A,* all-atom molecular dynamics simulation of cholesterol in liquid-disordered DPPC/DOPC/Chol membrane comparing cholesterol with phospholipid diffusion. *B,* Monte Carlo simulations of free, hop, and trapped diffusion probed by FCS showing how the hindrances affect the molecular diffusion. *Input* are the starting diffusion coefficients and *Output* are the diffusion coefficients when free diffusion or hindered diffusion (trapping or hopping) is employed in the simulations. *C,* statistical analysis of sFCS data showing that TF-PE can be fit with one component, whereas TF-SM and TF-Chol have two diffusional components; C1:slow component and C2:fast component. Mean ± S.D. of at least 10 independent simulations per condition is shown in *B*. In *C*, each data point shows a repetition of the sFCS measurement and each point consists of averaged data of >10 cells and >1000 curves. *Black lines* show the mean of the three repetitions.

To discern the role of hindrances in the plasma membrane on the slower diffusion of PE and SM analogues (and faster diffusion of cholesterol), we first simulated the diffusion of cholesterol and phospholipids *in silico* by molecular dynamics simulations in a ternary mixture of DPPC/DOPC/Chol, with the composition chosen to ensure a liquid-disordered membrane. Cholesterol diffusion was slightly faster (≈1.15 times) than phospholipids (Fig. 5*A*), which is in accordance with the experimental model membrane data (≈1.2 times, Fig. 2, *A–D*). (Note that the absolute diffusion coefficients vary between *in silico versus* the model membrane experiments due to a finite-size effect (42)).

We also simulated how the diffusion coefficients change when different hindrances are introduced (Fig. 5*B*) using Monte Carlo as described under “Experimental procedures.” For these simulations, we initialized the simulations with molecules having a diffusion coefficient of ≈1 μm^2^/s. We simulated molecules undergoing free, hop, and trapped diffusion modes. Although the simulations for all molecules were initialized with the same diffusion coefficients (that is, the microscopic diffusion constant in the absence of hindrances was 1 μm^2^/s), the diffusion coefficients of the molecules undergoing the hop and trapped diffusion yielded lower values showing that these hindrances actively slows down the diffusion of the molecules. Therefore, the slowed-down diffusion of SM, for instance, can be explained by diffusional hindrances.

To investigate the diffusion behavior of the TF-PE, TF-SM, and TF-Chol with high spatiotemporal and statistical accuracy, we performed scanning FCS statistical analysis. In this analysis, we generate histograms from all the FCS transit times and fit these with a log-normal distribution, which yields two parameters (μ and σ) (43). For a freely diffusing molecule, μ is closely related to the median transit time of the population (*e*^μ^ = median transit time) and σ accounts for the skew and spread of the distribution caused by the slow sampling in sFCS (Fig. S3). For molecules undergoing free diffusion, a simple log-normal fit represents the data well, unlike for molecules undergoing hindrance where a log-normal fit does not fit the data. In this case, a two-component (double lognormal) fit is required to describe the data, where the second component accounts for a fraction (1-B) of molecules with slower diffusion due to nanoscale interactions. We use the Bayesian Information Criterion for unbiased model selection (43–45) to decide between single and double log-normal distribution. Over the length scales accessible with this methodology, the data showed that TF-PE exhibits free diffusion with a single component with transit diffusion time (*e*^μ^) of 21 ± 2 ms (Fig. 5*C*). TF-SM showed two different diffusional components; slow component C1 with a diffusion time scale of *e*^μ^ ≈ 42 ± 13 ms and a fast component C2 with *e*^μ^ ≈ 25 ± 3 ms) (Fig. 5*C*, Fig. S3) which is a usual manifestation of trapped diffusion (as observed before with statistical analysis for Atto647N-SM (43)). The fast component (C2) of TF-SM is only slightly slower than TF-PE, which is in line with the model membrane data (see Fig. 2). The slow component (C1) was ≈2 times slower than TF-PE. Surprisingly, TF-Chol also showed two diffusional components (Fig. 5*C*). One component (*e*^μ^ ≈ 15 ± 6 ms) was slightly faster than TF-PE (≈1.4 times), a difference similar to what we observed in model membranes (see Fig. 2). The faster component (*e*^μ^ ≈ 9 ± 2 ms), however, was significantly faster (≈2.3 times) than TF-PE.

### Diffusion of fluorescent lipid analogues versus their localization

**Figure 6.**
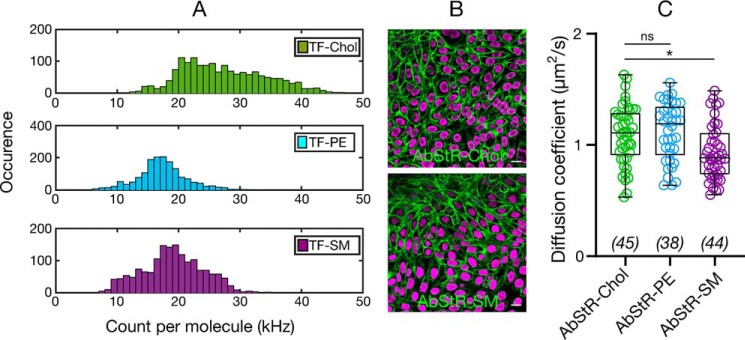
*A*, count per molecule (cpm) histograms determined from sFCS data for TF-Chol, TF-PE, and TF-SM in CHO cells showing higher cpm values for TF-Chol (>1000 curves). *B,* confocal images of CHO cells labeled with nonflipping Abberior Star Red-PEG-Chol and Abberior Star Red-SM. *Scale bars* are 10 μm. *C,* diffusion coefficient of Abberior Star Red-PEG-Chol, Abberior Star Red-PEG-PE, and Abberior Star Red-SM in CHO cells. Data are shown as box-and-whisker plot showing median, first and third quartiles, and all the data values. Number of data points are indicated on the graphs.

Two-component diffusion of TF-SM can be explained by its trapping behavior, which was observed previously with various methodologies and different SM analogues (38, 46–48). However, the two-component diffusion of cholesterol cannot be explained by simple trapping because one component is faster but not slower than the membrane diffusion of freely diffusing TF-PE. Moreover, previously TF-Chol has been reported to exhibit free diffusion (18, 19). It is postulated that the location of cholesterol (*e.g.* in different leaflet or its location relative to the membrane interface) in the membrane can drastically influence its diffusion (19). Leaflets in the plasma membrane are asymmetric; glycosphingolipids, sphingomyelin, and phosphatidylcholine are predominantly in the outer leaflet, whereas phosphatidylserine and phosphatidylethanolamine are mostly in the inner leaflet (49). This asymmetric composition creates a difference in the membrane organization, thus different microenvironments in the inner *versus* outer leaflets. Therefore, the fast diffusion of cholesterol compared with SM and PE in live cells may be due to their different leaflet preferences. There are contradictory reports on the leaflet preference of cholesterol; whereas some reports suggest outer leaflet enrichment of cholesterol (50), some recent reports contradict this hypothesis (51, 52), whereas others suggest more dynamic partitioning of cholesterol between leaflets (53). As inner and outer leaflets have different ordering (presumably with the inner leaflet being more fluid (54)), fast diffusion of cholesterol may be caused by differential partitioning of cholesterol. It was also previously speculated that alignment of cholesterol relative to the membrane plane alters its diffusion (19). Interestingly, recent studies suggested cholesterol localization in the mid-plane of the bilayer (between the leaflets) (55), which would heavily influence the diffusion of cholesterol. Therefore, we set out to address whether cholesterol localization in the membrane has an influence on its diffusion. First, we tested whether TF-Chol has different geometry inside the bilayer compared with TF-PE and TF-SM, which would be a strong indication of different localization. To this end, we used single molecule fluctuation analysis of sFCS data. Fluorophore alignment with respect to the membrane plane varies its extinction coefficient. The fluorophores are more efficiently excited (*i.e.* higher brightness) when the dipole moment of the fluorophore is parallel to the polarization of the laser. When we compared the brightness (photons per single particle) of the TF-PE, TF-SM, and TF-Chol in live cell membranes, we found that TF-Chol brightness was significantly higher than TF-PE and TF-SM, which suggests that TF-Chol geometry, and presumably localization in the membrane is different (Fig. 6*A*). To further test whether membrane localization has impact on diffusion, we used a cholesterol analogue, Abberior Star Red-PEG-Chol (AbStR-Chol), that cannot flip/flop in the membrane due to the PEG linker between the cholesterol and the dye and is located exclusively in the outer leaflet (29, 56). We compared its diffusion with Abberior Star Red-PEG-PE and Abberior Star Red-SM, which are also located in the outer leaflet. They stained the plasma membrane with no notable internal signal, which suggests that they do not flip to the inner part of the cell (Fig. 6*B*). The diffusion of AbStR-Chol, AbStR-PE, and AbStR-SM was approximately the same (≈1.0 μm^2^/s) and significantly slower than TF-Chol (≈2.4 μm^2^/s) indicating that cholesterol diffusion is as slow as phospholipid diffusion when located in the outer leaflet. Overall, these data suggests that cholesterol localization in the membrane is more complex than phospholipids and this localization influences its diffusion.

## Conclusion

Cholesterol fulfils major functions in cell biology by involving signaling and shaping cellular membrane structure. Building more quantitative models for the role of cholesterol in signaling demands a better understanding of its lateral diffusion. Here, we investigated the diffusion of fluorescent cholesterol analogues in both model and cellular membranes using advanced imaging and spectroscopy tools as well as molecular simulations. We found that cholesterol moves only slightly faster (≈1.2 times) than phospholipids and sphingolipids in model membranes that are thermodynamically in equilibrium. However, in live cells and embryos, it diffused significantly faster (≈2 times) than phospholipids and sphingolipids. We found that the slower diffusion of phospho- and sphingolipids (and thus faster diffusion of cholesterol) is not due to membrane domain partitioning. The fluorescent cholesterol analogue TF-Chol showed two diffusional components in live cells; one being similar to freely diffusing fluorescent PE analogue TF-PE and the other being faster. On the other hand, TF-SM also showed two diffusional components; one being similar to TF-PE, whereas the other being slower. This slow diffusional component of TF-SM is an indication of trapping (38). Brightness analysis revealed that the fast diffusional component of TF-Chol was due to heterogeneous localization/positioning of cholesterol within the membrane. This can be due to, for instance, asymmetric partitioning between the inner and outer leaflets of the plasma membrane and localization into the mid-plane of the bilayer (55, 57, 58). This is supported by the similar diffusion of cholesterol and phospholipid analogues when they are localized to the outer leaflet of the cell membrane.

Our results suggest an asymmetric localization and diffusion behavior of cholesterol in the membrane. These findings may be used to shed light on membrane asymmetry and heterogeneity. It will be intriguing to investigate the relationship between the complex diffusion behaviors of cholesterol and the membrane leaflet biophysical properties particularly in the context of membrane asymmetry. Moreover, our findings are crucial for membrane biology and naturally create new questions; how can cholesterol contribute to higher order membrane domain formation with sphingomyelin if their diffusion dynamics are so different and how can raft nanodomains be maintained? Are the fast and slow components of SM and Chol different and related to recently reported different forms of SM (59)? Do interactions occur between the fast SM and slow cholesterol, which have similar diffusion dynamics? What is the role of fast-moving cholesterol? Finally how can cholesterol contribute to cellular signaling with such fast diffusion dynamics? Many proteins have been proposed to have a cholesterol binding motif (3, 6, 8, 60, 61). It will also be essential to address how consensus cholesterol-binding motifs have been found in different membrane leaflets and whether it is related to cholesterol leaflet preference (62).

It is important to note that, here we use TF-Chol, a fluorescently-labeled cholesterol analogue that has been proven reliable for many aspects. However, there is still risk of artifacts induced by the fluorescent probe, thus further studies with label-free technologies will be useful to address these vital questions in cell biology. Furthermore, cholesterol localization and flipping may be critical for domain formation as suggested recently (63, 64). Thus, answers to these questions will also contribute to the efforts to understand the functional membrane heterogeneity. Our present measurement and experimental approaches give further guides to solving such long-standing questions.

## Experimental procedures

### Lipids and fluorescent lipid analogues

We purchased 23-(dipyrrometheneboron difluoride)-24-norcholesterol (TopFluor cholesterol; TF-Chol), 23-(dipyrrometheneboron difluoride)-24-norcholesteryl palmitate (16:0 TF-Chol), 23-(dipyrrometheneboron difluoride)-24-norcholesteryl linoleate (18:2 TF-Chol), 1,2-dioleoyl-sn-glycero-3-phosphocholine (DOPC), TopFluor-sphingomyelin (TF-SM), rhodamine PE, and TopFluor- phosphoethanolamine (TF-PE) from Avanti Polar Lipids. Abberior Star Red-PEG-cholesterol and Abberior Star Red-SM were obtained from Abberior. Cell Mask and NucBlue were obtained from Thermo Fisher Scientific.

### Preparation of GUVs

GUVs were prepared with the electroformation method as previously described (65). Briefly, a lipid film was formed on a platinum wire from 1 mg/ml of lipid mix (DOPC). Then, GUVs were formed in 300 mm sucrose solution at room temperature. 10 Hz, 2 V alternative electric current was used for electroformation.

### Cell culture and zebrafish embryos

CHO cells were maintained in Dulbecco's modified Eagle's medium/F-12 supplemented with 10% FBS medium and 1% l-glutamine.

For the zebrafish embryos, both female and male WT zebrafish strains were used. Breeding animals were between 3 months old and 2 years old. Zebrafish embryos that were used for the experiments were 24 h post fertilization. Animals were handled in accordance to procedures authorized by the UK Home Office in accordance with UK law (Animals (Scientific Procedures) Act 1986) and the recommendations in the Guide for the Care and Use of Laboratory Animals. All vertebrate animal work was performed at the facilities of Oxford University Biomedical Services. Adult fish were maintained as described previously (66). In brief, adult fish were exposed to 12 h light-12 h dark cycle (8 a.m. to 10 p.m. light; 10 p.m. to 8 a.m. dark), kept in a closed recirculating water system at 27–28.5 °C, fed 3–4 times a day, and kept at 5 fish per 1 liter density. Embryos were staged as described previously (67).

### GPMVs

GPMVs were prepared as previously described (34). Briefly, cells seeded on a 60-mm Petri dish (≈70% confluent) were washed with GPMV buffer (150 mm NaCl, 10 mm Hepes, 2 mm CaCl_2_, pH 7.4) twice. 2 ml of GPMV buffer was added to the cells. 25 mm Paraformaldehyde and 2 mm DTT (final concentrations) were added in the GPMV buffer. The cells were incubated for 2 h at 37 °C. Then, GPMVs were collected by pipetting out the supernatant. For phase-separated GPMVs, 20 mm DTT was used instead of 2 mm. To observe phase separation, cooling GPMVs to 10 °C may be necessary depending on the cell types.

### Fluorescent labeling of GUVs, GPMVs, cells, and zebrafish embryos

Tips of the pipette tips were cut before handling the GUVs to avoid GUV rupturing due to the shear stress. GUVs and GPMVs were labeled by adding the lipid analogues as well as Cell Mask and rhodamine PE to a final concentration of 10–50 ng/ml. For HDL labeling, 100 μl of vesicle suspension were incubated in 100 ng/ml of HDL (gift from Prof. Herbert Stangl) for 30 min. Labeled GUVs were placed in the wells of BSA-coated 8-well glass bottom Ibidi chambers that were filled with 250 μl of PBS.

For the cell labeling, the cells were seeded on 25-mm diameter round coverslips (number 1.5) in a 6-well-plate 2–3 days before the measurements. The fluorescent lipid analogues were first dissolved in DMSO or ethanol with a final concentration of 1 mg/ml. Before the labeling, the cells seeded on glass slides were washed twice with L15 medium to remove the full medium. Please note that serum in the media decreases the labeling efficiency, thus it is crucial to wash out all the media from the cells. Later the fluorescent analogues were mixed with L15 medium with 1:1000 ratio (final concentration of 1 μg/ml). The cells were incubated with this suspension for 5–10 min at room temperature. After that, the cells were washed with L15 twice followed by imaging in the same medium. Then, confocal microscopy was performed as described below. Labeling cells with fluorescent analogues should be optimized for every cell line by changing the concentration, labeling time, and labeling temperature. For nucleus labeling, NucBlue live nucleus staining was done. One drop of NucBlue was added in 1 ml of medium and the cells were incubated in this solution for 15 min.

Zebrafish embryos (24 hpf) were incubated in E3 buffer (4.5 mm NaCl, 0.18 mm KCl, 0.33 mm CaCl_2_·2H_2_O, 0.4 mm MgCl_2_·6H_2_O in water) for 24 h and dechorionated manually using forceps. Later, they were mixed with lipid analogues (final concentration of 0.5 μg/ml) for 1 h at 28 °C followed by another 1 h of a gentle nutation at room temperature. The embryos were transferred to a 10-cm Petri dish filled with fresh E3 solution for washing. Later, the embryos were washed twice with E3 medium and transferred to the Ibidi chambers filled with 250 μl of E3 buffer for imaging.

### Confocal microscopy

GUVs were imaged in PBS, GPMVs were imaged in GPMV buffer, cells were imaged in L15 medium, and embryos were imaged in E3 buffer. All imaging was done at room temperature (21–23 °C) to avoid analogue internalization (see Fig. S4 for expected internalization). All imaging was done on glass slides with thickness of 0.17 mm. Samples were imaged with a Zeiss LSM 780 (or 880) confocal microscope in BSA-coated (1 mg/ml for 1 h) 8-well Ibidi glass chambers (number 1.5). TF-labeled analogues were excited with 488 nm and emission was collected between 505 and 550 nm. Abberior Star Red-labeled analogues as well as Cell Mask Deep Red were excited with 633 nm and emission collected with 650–700 nm. NucBlue was excited with 405 nm and emission was collected at 420–470 nm. Multi-track mode was used to avoid cross-talk.

### FCS

FCS on the GUVs and GPMVs were carried out using Zeiss LSM 780 (or 880) microscope, 40× water immersion objective (numerical aperture 1.2) as described before (48). Briefly, before the measurement, the shape and size of the focal spot was calibrated using Alexa 488 and Alexa 647 dyes in water in an 8-well glass bottom (number 1.5) chamber. This optical setup yielded full-width at half-maximum of 250 nm and 0.06–0.07 μm^2^ observation area in lateral dimension. Because the membrane diffusion is restricted to two dimensions, the size of the lateral dimensions were used to calculate the diffusion coefficients.

To measure the diffusion on the membrane, GUVs and GPMVs were placed into an 8-well glass bottom (number 1.5) chamber coated with BSA. The laser spot was focused on the top membrane by maximizing the fluorescence intensity. Then, 3–5 curves were obtained for each spot (5 s each). Cell measurements were performed at the basal membrane of the cells. The laser spot was focused on the bottom membrane by maximizing the fluorescence intensity. To avoid the cross-talk from internalized fluorophores, measurements were done at the bottom of the nucleus (Figs. S2 and S4). For zebrafish embryos, similarly the laser focus was placed on the membrane by maximizing the fluorescence signal. 3–5 curves were obtained for each spot (5 s each).

The obtained curves were fit using the freely available FoCuS-point software (68) using 2D and triplet model. Please see exemplary point FCS curves in Fig. S5 and their fits in Fig. S6.

All scanning FCS experiments were performed on the Zeiss LSM 780 using the 40× 1.2 NA FCS water objective as described previously (43). TF fluorescence was excited using the 488 Argon laser passing through a 488/594/633 MBS and fluorescence was detected using the hybrid GaAsP detector (photon counting mode) in the 500- to 600-nm range. All sFCS measurements were obtained as line scans with a length of 5.2 μm (52 pixels with a pixel size of 100 nm) at the bottom membrane of live CHO cells right below the nucleus. Maximum scanning frequency (2081 Hz) was used and data were acquired for 50 s. The scans were correlated with the FoCuS-scan software (69, 70). For all data sets the first 10 s were cropped off to remove initial bleaching and an 18-s photobleaching correction by local averaging applied. The correlation curves were fitted including 10 times bootstrapping with a 2D one-component model (no anomalous subdiffusion, no triplet etc.) and the resulting fitting parameters (transit time, counts per molecule) were exported for further analysis. Please see exemplary scanning FCS curves in Fig. S5.

### Scanning FCS statistical analysis

Statistical analysis of sFCS data were performed as described previously (43). Briefly, the exported transit times from the FCS fits were histogrammed using Matlab. The resulting transit time histograms were fitted with a log-normal function (for single component/free) or with a double log-normal function (for hindered diffusion). Model selection was performed using maximum likelihood estimation and employing the Bayesian Information criterion. To obtain increased fitting accuracy the data were cumulatively, linearly, and logarithmically histogrammed and fitted with the respective log-normal function. Thus a large data set of >10 cells with multiple measurements each resulting in >1000 curves for one repetition can be summarized in the statistical analysis fitting parameters.

### % Lo calculation

ImageJ-Line profile was used to calculate the Lo % as described in Ref. 14. A line was selected that crosses the opposite sides of the equatorial plane of the GPMVs having different phases on opposite sides. Opposite sides are chosen to eliminate the laser excitation polarization artifacts. Then, % Lo was calculated as,

(Eq. 1) $$\%\;Lo=\frac{I_{Lo}}{I_{Lo}+I_{Ld}}$$

where *I* is the fluorescence emission intensity. If % Lo > 50%, a lipid analogue prefers the liquid-ordered phase.

### Molecular simulations of DPPC/DOPC/Chol membranes

240 DOPC, 116 DPPC, and 44 CHOL per leaflet were assembled into a bilayer and solvated with 40 TIP3P waters using the CHARMM-GUI (71) membrane builder. The system was equilibrated at a constant temperature of 298 K and constant pressure of 1 bar using semi-isotropic pressure coupling with NAMD version 2.7 on local resources for 50 ns. The system was then transferred to the Anton special purpose supercomputer (72) for production simulation. The equations of motion were integrated with the Verlet algorithm with a time step of 2.0 fs. A constant temperature and a pressure of 1 atm were maintained by the Martyna-Tobias-Klein (73) method, with the pressure coupling effected every 240 fs and the temperature coupling every 24 fs. Lennard-Jones interactions were truncated at 10.14 Å by a hard cutoff with no shift. Long-range electrostatics were computed by the *k*-space Gaussian split Ewald method (74) on a 64 Å ∼ 64 Å ∼ 64 point grid, with the parameters of the Gaussian chosen to yield a root mean square error in the electrostatic force calculation of 0.18%. The total duration of the production simulation was 1.2 μs. Full simulation details can be found in Ref. 75.

### Monte Carlo simulations of diffusion modes

For the simulation of free and hindered diffusion we used and slightly modified the nanosimpy python repository, as described previously (48). 350 particles were randomly initiated and moved at every time step (1 μs) according to having a diffusion coefficient of 1 μm^2^/s. Diffusion was simulated for 20 s on a circle with a diameter of 3 μm (wrapped around on the edges). A FCS observation spot (approximated by a Gaussian) was placed in the center of the simulation area and the apparent intensity sampled at every time step. The observation size was 250 nm. For trapped diffusion we employed a statistical model for molecular complex formation rending a random particle immobile for a short time (p_trap_ = p_untrap_ = 0.00005). For hop diffusion we generated a Voronoi mesh with a characteristic mesh size (given as √) of 110 nm. When a particle would hit a boundary it only had a low chance (phop = 0.05) to pass on to the next compartment and otherwise bounce off. FCS curves were fitted with the FoCuS_point software using a simple 2D diffusion model (68). Note that for the case of hindered diffusion modes anomalous subdiffusion can occur (meaning the α-value can drop below 1, ranging from 0.65 to 1).

## Author contributions

K. P., F. S., M. L., and E. S. investigation; K. P., F. S., M. L., E. L., and E. S. methodology; F. S., E. L., and E. S. software; F. S., E. L., C. E., and E. S. writing-review and editing; M. L., T. S.-S., E. L., and E. S. resources; T. S.-S., C. E., and E. S. supervision; E. L. and E. S. visualization; C. E. and E. S. funding acquisition; C. E. and E. S. writing-original draft; E. S. conceptualization; E. S. project administration.

## Supplementary Material

Supporting Information

## Footnotes

2 The abbreviations used are:

Chol: cholesterol
GUV: giant unilamellar vesicles
GPMV: giant plasma membrane vesicles
TF: TopFluor
PE: phosphatidylethanolamine
SM: sphingomyelin
CHO: Chinese hamster ovary
FCS: fluorescence correlation spectroscopy
AbStR: Abberior Star Red
HDL: high density lipoprotein
Lo: liquid-ordered
sFCS: scanning FCS
DOPC: 1,2-dioleoyl-*sn*-glycero-3-phosphocholine
DPPC: 1,2-dipalmitoyl-*sn*-glycero-3-phosphocholine.
