# Supplementary material for: Nanoscale dynamics of cholesterol in the cell membrane
Source: J Biol Chem. 2019 Jul 3;294(34):12599–609. doi: 10.1074/jbc.RA119.009683 (PMC6709632; doi:10.1074/jbc.RA119.009683)
Supplement: Supporting Information [file supp_294_34_12599__index.html]

Nanoscale dynamics of cholesterol in the cell membrane — Nanoscale cholesterol diffusion — Nanoscale dynamics of cholesterol in the cell membrane — Nanoscale cholesterol diffusion — Supporting Information 

# Nanoscale dynamics of cholesterol in the cell membrane

## Supporting Information

- Supporting Information (to be published online) - Supporting Figures (Figures S1-S6)
