# Supplementary material for: Nanoscale dynamics of cholesterol in the cell membrane
Source: J Biol Chem. 2019 Jul 3;294(34):12599–609. doi: 10.1074/jbc.RA119.009683 (PMC6709632; doi:10.1074/jbc.RA119.009683)
Supplement: Supporting Information [file supp_RA119.009683_153363_1_supp_354046_ptpw10.pdf]

Supplementary Information

Supplementary Figures

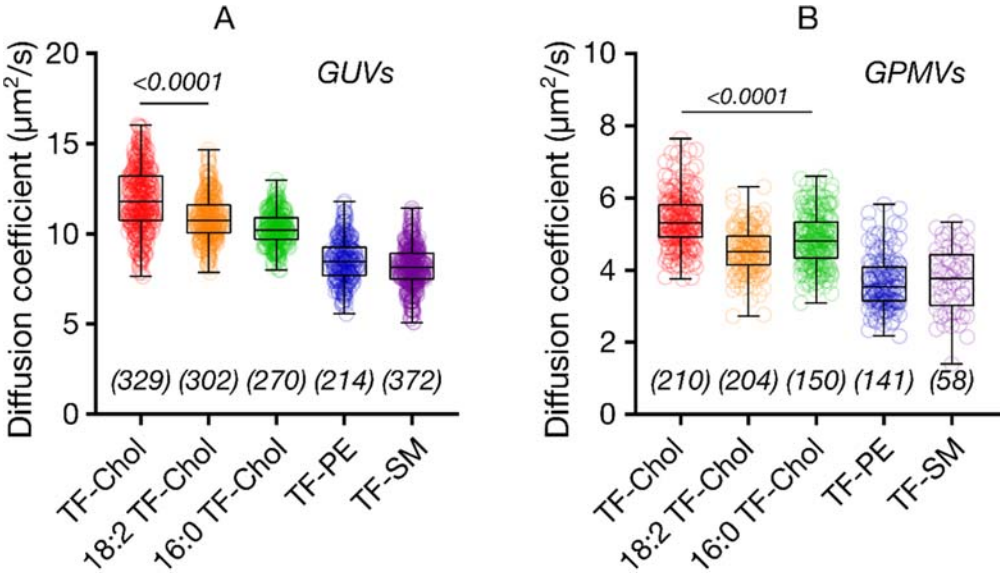

Figure S1. Diffusion of cholesterol analogue TF-Chol compared to phospholipid and sphingolipid analogues in model membranes in A) GUVs and B) GPMVs. Data is pooled from at least three independent measurements. Data are shown as box-and-whisker plot showing median, first and third quartiles, and all the data points. Number of data points are indicated on the graphs.

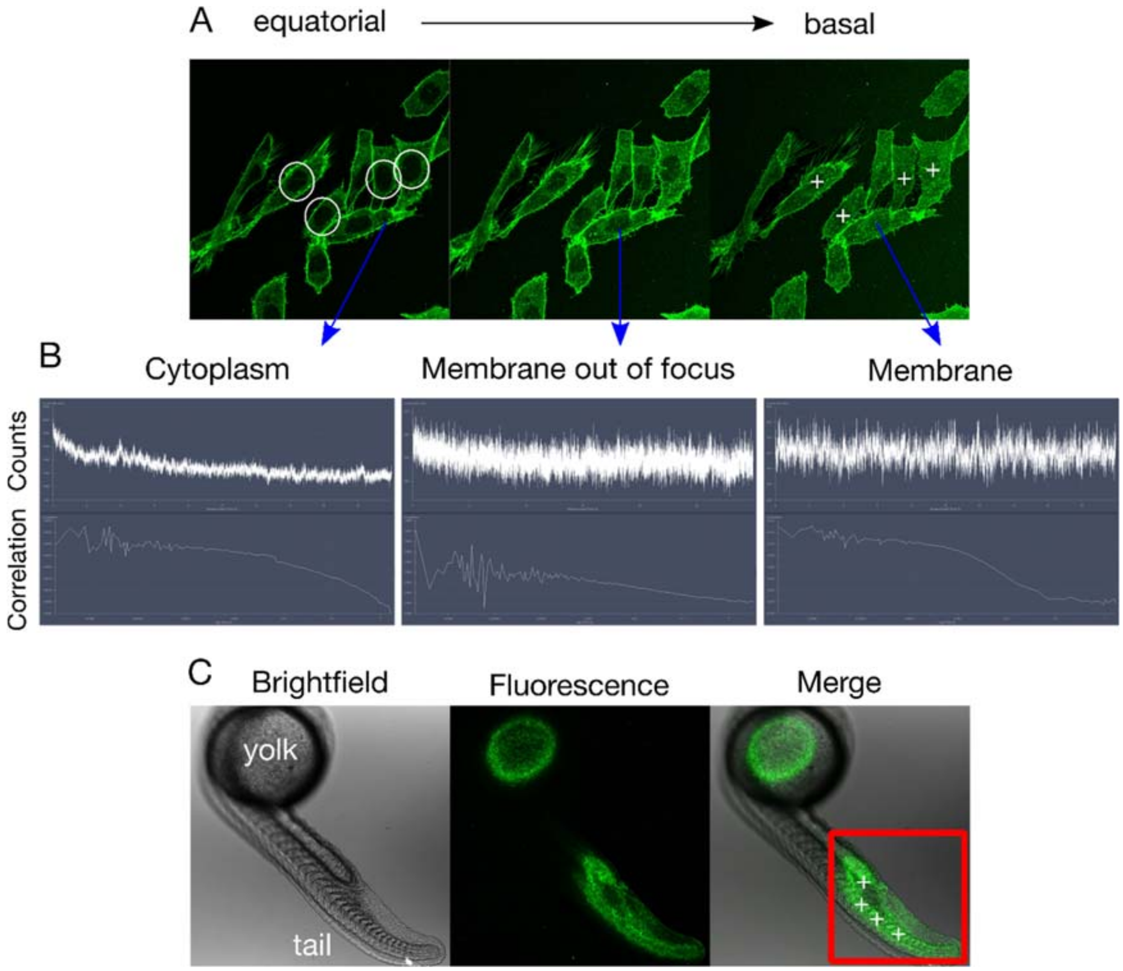

Figure S2. Position selection for FCS measurements in A, B) live cells and C) embryos labelled with TF-SM. A) To avoid internal background, we performed the FCS measurements on the bottom membrane underneath the nucleus where there is no internal background. B) When the measurement is taken from the cytoplasmic region, strong bleaching is observed followed by low quantum yield fluctuations. When membrane is slightly out of focus (i.e., the measurement is taken 500 nm above the membrane plane towards the cytoplasm), the transit time gets longer, count per particle goes down and quality of the curve decreases. The counts and counts per particle are the highest when the membrane is in perfect focus. This shows that our measurements are free from artefacts that may result from internal signal. C) For zebrafish embryos, we measured FCS close to the tail region away from the yolk which accumulated lipid analogues.

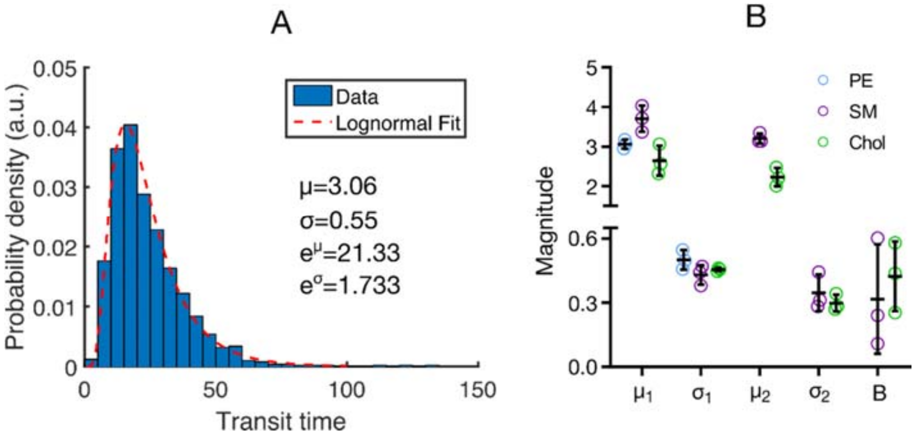

Figure S3. A) Simulated lognormal distribution of sFCS transit time data and the respective fitting parameters for free diffusion with an apparent transit time of 21.33 ms. B) sFCS fitting parameters obtained from the lognormal fit for the three fluorescent lipid analogues. TF-PE histograms can be fitted with a single lognormal function whereas a double lognormal fit needs to be employed for TF-SM and TF-Chol.

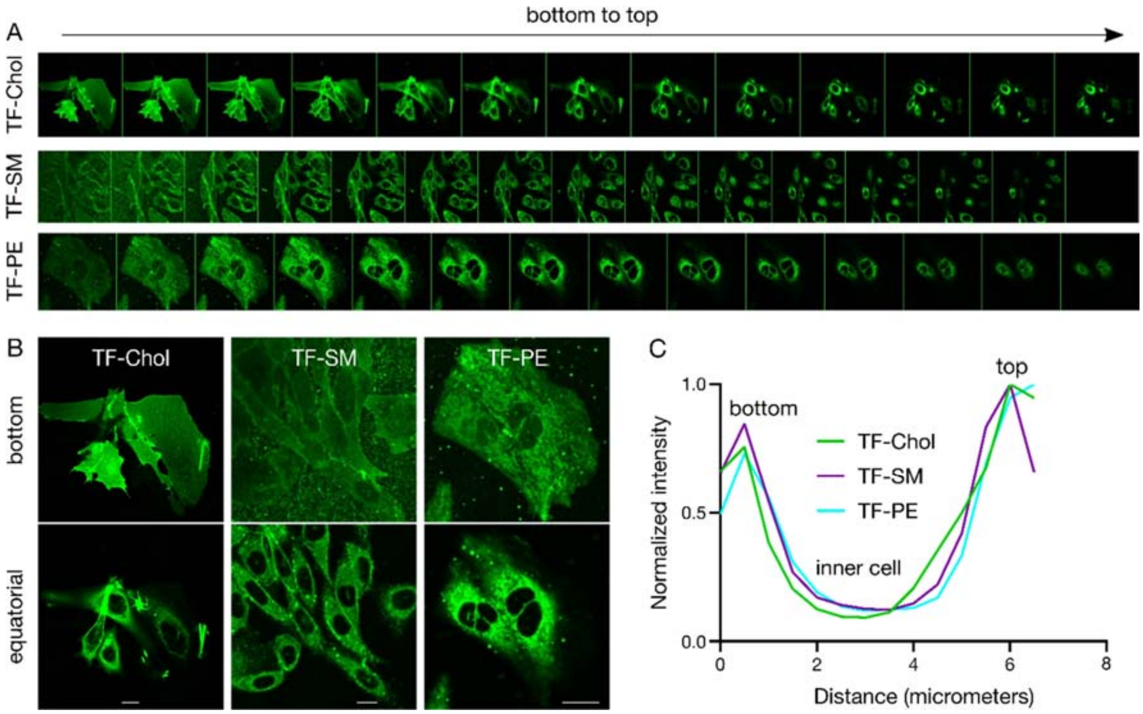

Figure S4. A) Optical sectioning of cells labelled with TF-Chol (top), TF-SM (middle) and TF-PE (bottom). Each axial step is 500 nm. B) Images of bottom and equatorial planes of cells labelled with fluorescent lipid analogues. This indicates minimum internalization for TF-Chol and TF-SM and intermediate internalization for TF-PE. Scale bars are 10 µm. C) z-axis intensity profile obtained from a line along the axial direction spanning from basal membrane underneath the nucleus to top membrane. Intensity is maximum at the top and the bottom membrane for all the probes. This again shows that there is not notable disturbance from the internal structures when the measurement is done on the plasma membrane underneath the nucleus.

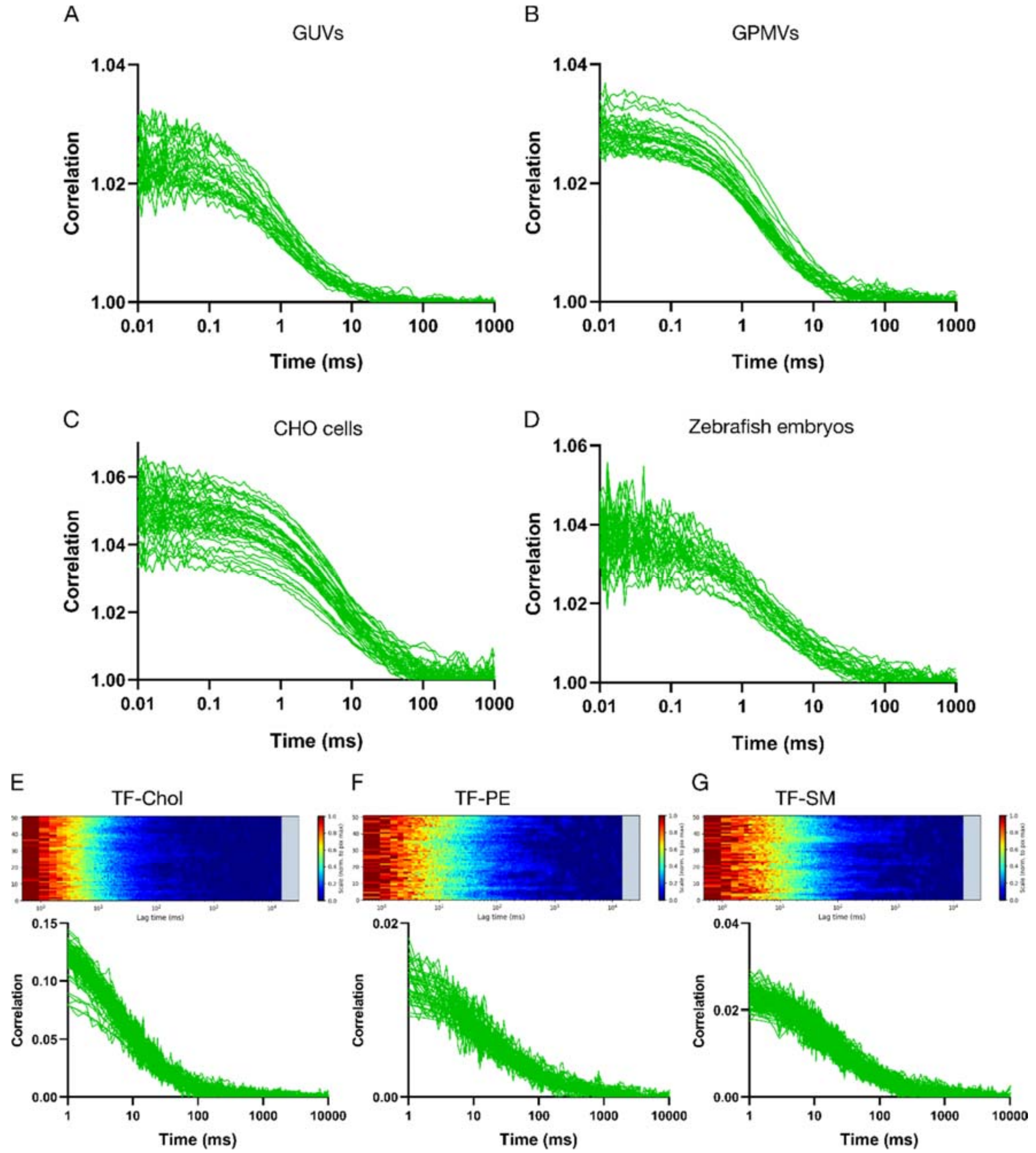

Figure S5. Exemplary FCS curves of TF-Chol in A) GUVs B) GPMVs C) CHO cell and D) zebrafish embryos. E, F, G) Exemplary scanning FCS carpets (top) and resulting FCS curves from these carpets (bottom) in CHO cells labelled with E) TF-Chol, F) TF-PE and G) TF-SM.

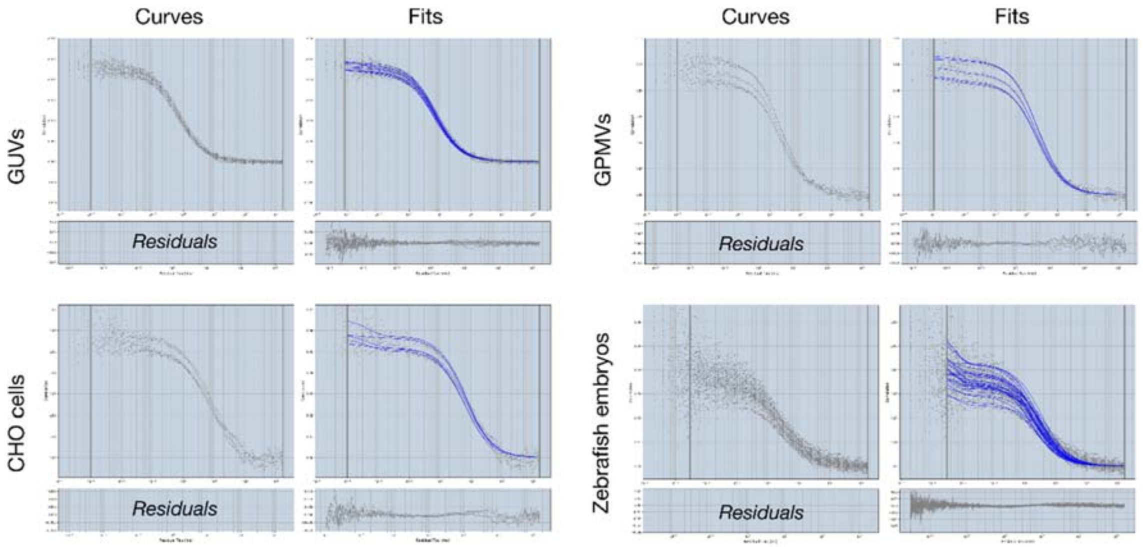

Figure S6. Exemplary FCS curves and their fits in GUVs, GPMVs, CHO cell and zebrafish embryos labelled with TF-Chol. Residuals around 0 confirms the quality of the fits.
